# Supplementary material for: Modeling glioblastoma heterogeneity as a dynamic network of cell states
Source: Mol Syst Biol. 2021 Sep 16;17(9):e10105. doi: 10.15252/msb.202010105 (PMC8444284; doi:10.15252/msb.202010105)
Supplement: Supplementary file 6 — Source Data for Figure 5 [file MSB-17-e10105-s004.zip › Figure5A_sourcedata/GSEA_3017/nosigdb_stateB.GseaPreranked.1622795021725/RICHARDS_NOWAKOWSKI_SCIENCE_2017_NIN1_UPREG.html]

Details for gene set RICHARDS\_NOWAKOWSKI\_SCIENCE\_2017\_NIN1\_UPREG[GSEA]

|  || Dataset | state43017 |
| Phenotype | NoPhenotypeAvailable |
| Upregulated in class | na\_neg |
| GeneSet | RICHARDS\_NOWAKOWSKI\_SCIENCE\_2017\_NIN1\_UPREG |
| Enrichment Score (ES) | -0.49064723 |
| Normalized Enrichment Score (NES) | -2.291392 |
| Nominal p-value | 0.0 |
| FDR q-value | 0.0038274578 |
| FWER p-Value | 0.007 |
Table: GSEA Results Summary

  

Fig 1: Enrichment plot: RICHARDS\_NOWAKOWSKI\_SCIENCE\_2017\_NIN1\_UPREG      
 Profile of the Running ES Score & Positions of GeneSet Members on the Rank Ordered List

  

| PROBE | GENE SYMBOL | GENE\_TITLE | RANK IN GENE LIST | RANK METRIC SCORE | RUNNING ES | CORE ENRICHMENT || 1 | NCAM1 |  |  | 178 | 0.436 | -0.1558 | No |
| 2 | CCNE2 |  |  | 215 | 0.406 | -0.1266 | No |
| 3 | GRIA4 |  |  | 358 | 0.322 | -0.2557 | No |
| 4 | NAV3 |  |  | 534 | 0.275 | -0.4381 | Yes |
| 5 | ALYREF |  |  | 546 | 0.273 | -0.4008 | Yes |
| 6 | CDH2 |  |  | 558 | 0.271 | -0.3639 | Yes |
| 7 | ST8SIA5 |  |  | 588 | 0.266 | -0.3520 | Yes |
| 8 | DCX |  |  | 689 | -0.264 | -0.4358 | Yes |
| 9 | PLS3 |  |  | 700 | -0.275 | -0.3967 | Yes |
| 10 | CHD7 |  |  | 706 | -0.284 | -0.3492 | Yes |
| 11 | SOX4 |  |  | 712 | -0.305 | -0.2976 | Yes |
| 12 | FAM107B |  |  | 737 | -0.416 | -0.2505 | Yes |
| 13 | BTG1 |  |  | 741 | -0.423 | -0.1739 | Yes |
| 14 | TUBB3 |  |  | 747 | -0.448 | -0.0951 | Yes |
| 15 | SOX11 |  |  | 754 | -0.576 | 0.0067 | Yes |
Table: GSEA details [plain text format]

  

Fig 2: RICHARDS\_NOWAKOWSKI\_SCIENCE\_2017\_NIN1\_UPREG: Random ES distribution      
 Gene set null distribution of ES for **RICHARDS\_NOWAKOWSKI\_SCIENCE\_2017\_NIN1\_UPREG**
